# Premenstrual dysphoric disorder and its co-existence with depression, anxiety, and stress as risk factors for suicidal ideation and suicide attempts among university students in Bangladesh: A single-site survey

**DOI:** 10.1371/journal.pone.0321097

**Published:** 2025-04-01

**Authors:** Nitai Roy, Sultan Mahmud Imran, Aysha Siddiky, Samia Sultana, Sumana Mahmud, Rejwana Rashid, Moneerah Mohammad ALmerab, Mohammed A. Mamun

**Affiliations:** 1 Department of Biochemistry and Food Analysis, Patuakhali Science and Technology University, Patuakhali, Bangladesh; 2 Faculty of Nutrition and Food Science, Patuakhali Science and Technology University, Patuakhali, Bangladesh; 3 Department of Psychology, College of Education and Human Development, Princess Nourah Bint Abdulrahman University, Riyadh, Saudi Arabia; 4 CHINTA Research Bangladesh, Dhaka, Bangladesh; 5 Department of Public Health and Informatics, Jahangirnagar University, Dhaka, Bangladesh; 6 Department of Public Health, University of South Asia, Dhaka, Bangladesh; University of the Basque Country UPV / EHU, SPAIN

## Abstract

**Background:**

Premenstrual Syndrome (PMS) and Premenstrual Dysphoric Disorder (PMDD) have been identified as potential risk factors for various mental health issues, such as suicidal ideation and attempts. However, few studies have examined this association among Bangladeshi university students. This study aimed to examine the potential associations between PMDD and its co-existence with depression, anxiety, stress, and various suicidal behaviors, including suicidal ideation and attempts.

**Methods:**

A cross-sectional survey involving 516 university students was conducted between September and October 2023. The survey was carried out in person and employed a structured questionnaire that comprised demographic information; the Depression, Anxiety, and Stress Scale (DASS-21); and the Premenstrual Symptoms Screening Tool (PSST), a 19-item screening tool for premenstrual symptoms. Multivariable logistic regression analyses were conducted to examine the relationships between variables.

**Results:**

In the present study, participants with PMDD reported a prevalence of 38.8% for past-year suicidal ideation and 28.6% for suicide attempts. Through logistic regression analysis, we found a significant association between moderate/severe PMS and PMDD and a higher likelihoods of reporting suicidal ideation (AOR =  4.73; 95% CI 2.08–10.73 and AOR =  5.42; 95% CI 2.02–10.52) and suicide attempts (AOR =  3.77; 95% CI 1.36–10.50 and AOR =  4.07; 95% CI 1.22–15.56). The association between suicidal behaviors and PMS/PMDD was mediated by co-existing conditions such as depression, anxiety, and stress.

**Conclusions:**

A notable proportion of individuals diagnosed with PMDD reported experiencing suicidal ideation or engaging in suicide attempts at some point in their lives. The findings of this research support the importance of conducting regular assessments of suicidal risk among women experiencing moderate to severe premenstrual disturbance. Furthermore, it is crucial to integrate mental health screenings and implement psychosocial interventions specifically designed for women diagnosed with PMS or PMDD and those with co-existing depression, anxiety, and stress alongside PMS/PMDD.

## Introduction

Suicide is a substantial public health concern that transcends geographical boundaries and affects populations worldwide. According to the World Health Organization, approximately total of nearly 800,000 suicide deaths occur annually. This represents the frequency of one suicide occurring approximately every 40 seconds [1,2]. However, age-specific mortality indicates that suicide is the second most prevalent cause of mortality among individuals aged 10–14 and 20–34 [3]. Concerning gender-specific suicidality, it has appeared that females are more likely to attempt suicide, but actual suicide mortality is higher in males [4,5]. However, the complex nature of suicide encompasses a multitude of factors, including biological, social, and psychological elements, which explain the completion of suicidality pathways and actions of suicide. It has been claimed that more than 90% of suicide cases are directly and/or indirectly related to psychiatric suffering [6], which includes depression [7], anxiety disorder [8], posttraumatic stress disorder [9], insomnia, obsessive-compulsive disorder [10], and schizophrenia [11].

Premenstrual syndrome (PMS) is a collection of physical, emotional, and behavioral symptoms experienced by many women. These symptoms can range from mild to severe and have a significant impact on daily life, often causing disruptions in work and personal activities [12]. Premenstrual Dysphoric Disorder (PMDD) represents a heightened manifestation of PMS, specifically characterized by more intense depressive and anxiety-related symptoms [13]. Both PMS and PMDD typically subside within a few days after menstruation. PMS and PMDD impact a significant percentage of women, with estimates ranging from 75% to 85% for PMS [14] and 5% to 8% for PMDD globally [15]. The International Classification of Diseases (ICD-10) only requires the presence of one distressing symptom during the premenstrual phase to diagnose PMS [16]. On the other hand, the Diagnostic and Statistical Manual of Mental Disorders (DSM-5) has specific criteria for diagnosing PMDD [17]. These criteria include the presence of at least five symptoms during the final week before the onset of menstruation, such as marked affective lability, irritability, anger, depression, decreased interest in activities, difficulty concentrating, increased fatigue, changes in appetite and sleep, and effects on work, school, relationships, responsibilities, and social activities.

Physiological and psychological symptoms associated with menstruation can potentially influence mental health outcomes. Dysmenorrhea (physical pain and discomfort), a prevalent occurrence among the female population (45% to 95% of studies), is associated with diminished perceptions of health-related quality of life and reduced self-esteem [18,19]. In addition, menstruation, together with hormonal variations during the luteal and premenstrual stages, is linked to heightened anxiety, dysphoria, and mood problems [20–22]. Research demonstrates that although menstruation may induce emotional distress, the premenstrual period, especially during the late luteal phase, is more significantly associated with increased psychological symptoms and social withdrawal [23]. The presence of behavioral symptoms, such as a decrease in social interaction during the menstrual cycle, may potentially contribute to the development of feelings of isolation and depression [24]. The potential correlation between psychological symptoms and mental health outcomes during menstruation implies that the symptoms encountered during this phase could contribute to significant mental health consequences.

Saunders and Hawton (2006) conducted a comprehensive review of 44 studies on the relationship between suicide and the menstrual cycle, revealing a significant correlation between PMS and suicide attempts in women [25]. The study, based on three specific papers, identified PMS as a risk factor for suicidal behaviors, indicating that women experiencing PMS should be considered at a heightened risk of suicidal behaviors. According to a recent systematic literature review, there is a robust association between PMDD and suicidal thoughts, ideation, plans, and attempts [26]. This association remained significant even after controlling for the presence of co-occurring psychiatric conditions. Nevertheless, it was shown that women diagnosed with PMDD did not exhibit more pronounced risk factors for suicide attempts or a higher frequency of attempts during the luteal phase of the menstrual cycle, in comparison to individuals who had attempted suicide but did not have PMDD [26]. According to a meta-analysis, it has been found that PMDD is a significant and robust predictor of suicidality [27]. The investigation revealed that those with PMDD were nearly seven times more likely to attempt suicide and four times more likely to experience suicidal thoughts than those without PMDD. It is noteworthy that although prior research has established associations between PMS or PMDD and suicidality, there remains a dearth of studies examining these conditions within the student community, which is considered a high-risk category for suicide in Bangladesh [28].

This study aims to fill the existing gaps by investigating the association between PMDD and its co-existence with depression, anxiety, stress, and suicidality (ideation and attempts) in a sample of university students in Bangladesh. Based on previous investigations, our research team formulated a hypothesis suggesting that students who experience PMDD, but not those with moderate to severe PMS, would exhibit a significantly higher likelihood of reporting an occurrence of suicidal behaviors than students who do not have PMDD or moderate/severe PMS.

## Methods

### Participants and procedure

A cross-sectional survey was conducted between September and October 2023 among female students enrolled at Patuakhali Science and Technology University in Patuakhali, Bangladesh. The survey employed a convenience sampling approach, targeting students in the classroom who willingly consented to participate. This approach was selected because of its logistical feasibility and accessibility to participants within the university setting. Face-to-face interviews were conducted to collect data from 530 students. After excluding incomplete questionnaires, a total of 516 participants completed the survey, resulting in a response rate of 97.4%.

### Ethical clearance

The research protocol was reviewed and approved by the Research Ethical Committee (REC) of the Department of Biochemistry and Food Analysis at Patuakhali Science and Technology University in Bangladesh. The assigned approval number for this protocol is PSTU/IEC/2023/65 (14). All participating students exhibited comprehension of the nature, objectives, procedures, and handling of the gathered data. Before the commencement of the research, written consent was obtained from the participants, ensuring that no harm would result from their involvement. Consent documents were duly recorded and stored according to the research protocol. The confidentiality and anonymity of the participants’ personal information were assured throughout the study. Moreover, the study participants were informed of their rights regarding participation, data confidentiality, and the option to withdraw their data, following the principles outlined in the Helsinki Declaration of 1975.

### Instruments

#### Socio-demographic and menstruation-related variables.

This study examined various socio-demographic factors, including age, religion, marital status, physical exercise, and chronic illness. The participants’ physical activity levels were assessed through self-reporting, in which they were asked if they engaged in at least 30 minutes of moderate-intensity physical activity daily that increased their heart rate or breathing, as described in a previous study [29]. Menstrual variables, such as age at menarche, family history of premenstrual symptoms (simple ‘yes/no’), and academic performance impairment due to premenstrual symptoms (simple ‘yes/no’), were also incorporated in the study. Additionally, the prevalence of chronic diseases among participants was evaluated by inquiring about their medical history of hypertension, diabetes, cardiovascular disease, cancer, stroke, and chronic obstructive pulmonary disease during the survey administration.

#### Premenstrual symptoms and premenstrual dysphoric disorder.

A modified version of the Premenstrual Symptoms Screening Tool (PSST) was used to assess the presence and severity of premenstrual symptoms [30]. The PSST consists of 19 items, including 14 premenstrual symptoms and five functional items, aligned with the DSM-IV criteria. The study involved evaluating participants’ symptom experiences and functional interference during the late luteal phase, with ratings obtained for each symptom and functional domain until a few days before the onset of menstruation. The items were rated on a scale of “not at all,” “mild,” “moderate,” or “severe.”

The PSST was organized into three categories to effectively diagnose PMDD and severe PMS: “core PMS” symptoms, “other PMS” symptoms, and “functional” items. For the diagnosis of PMDD, the criteria included at least one of four severe “core PMS” symptoms, at least four additional moderate or severe PMS symptoms, and at least one of five severe “functional” items. The diagnosis of severe PMS followed similar criteria but with a less stringent threshold. It required the presence of at least one of the four core symptoms rated as moderate or severe, at least four additional moderate or severe PMS symptoms, and at least one of the five functional items rated as moderate or severe. The objective of categorizing “severe PMS” was to identify adolescent females experiencing clinically significant PMS symptoms that did not meet the stringent criteria for PMDD. The reliability of the PSST questionnaire was found to be high, with Cronbach’s Alpha coefficients of 0.91 for the total of 19 items, 0.89 for the first 14 items, and 0.88 for the last 5 items, as validated and verified in this study.

### Depression, anxiety, and stress

The DASS-21 is a self-report questionnaire designed to assess levels of depression, anxiety, and stress [31]. The survey consists of three subscales, each consisting of seven questions. Participants rated each item on a 4-point scale, ranging from 0 (indicating that the statement did not apply to them at all) to 3 (indicating that it applied to them very much or most of the time). The original scores were multiplied by two to obtain the final score. These scores were categorized into five groups: normal, mild, moderate, severe, and extremely severe. For the normal group, the depression, anxiety, and stress scores ranged from 0 to 9, 0–7, and 0–14, respectively. The mild depression, anxiety, and stress scores were in the ranges of10–13, 8–9, and 15–18, respectively. Moderate depression, anxiety, and stress scores were within the ranges of 14–20, 10–14, and 19–25, respectively. The severe group exhibited depression, anxiety, and stress levels ranging from 21 to 27, 15–19, and 26–33, respectively. Finally, the extremely severe group had depression, anxiety, and stress scores of 28 or higher, 20 or higher, and 34 or higher, respectively [31]. The reliability of the DASS-21, as measured by Cronbach’s alpha coefficients, was 0.93 for depression, 0.87 for anxiety, 0.84 for stress, and 0.86 for overall measures. These coefficients indicated excellent and good reliability for the overall scale and individual subscales, respectively.

### Suicidal ideation and suicide attempts

Past-year suicidal ideation and suicide attempts were evaluated as binary variables. Consistent with prior research [32,33], participants were asked if they had experienced suicidal ideation or attempted suicide in the past year. A binary coding system was utilized, where a “yes” response was coded as a “1” and a “no” response was coded as a “0.”

### Statistical analyses

Data analysis was performed using SPSS version 28.0. Initially, descriptive statistics such as frequencies, percentages, mean values, and standard deviations were used to present the data. To investigate the relationship between the independent variables and outcome variables (suicidal ideation and suicide attempts), a chi-square test was employed. Before conducting binary regression analyses, a multicollinearity assessment was performed. The models in this study included all relevant variables, including the dependent variables of past-year suicidal ideation and suicide attempts. The fit of the was evaluated using the Hosmer-Lemeshow test, with a significance level of *p* ≥  0.05; p values for suicidal ideation and suicide attempt were 0.630 and 0.722, respectively. Linear regression analysis was employed to examine the interaction effects of PMS/PMDD with depression, anxiety, and stress. Standardized beta coefficients (β) were computed to assess the strength and direction of relationships. The analysis was conducted with a 95% confidence interval, and the significance level was set at *p* < 0.05.

## Results

### Sample description

The mean age of the participants was 21.71 (SD =  10.85) years. Among the respondents, 48.4% were aged ≤ 21 years, 90.7% were single, and 59.9% were Muslim. Of the total sample size, 87.6% reported no chronic conditions, while 68.8% reported not engaging in regular physical activity. However, the prevalence rates of mild, moderate, severe, and extremely severe depression were 15.1%, 21.9%, 18.4%, and 13.2%, respectively, which was 8.5%, 29.1%, 10.7%, and 17.6%, and 13.6%, 16.1%, 14.5%, and 5.0%, respectively, for anxiety and stress.

In terms of menarche age, the majority of the participants (70.5%) reported experiencing menarche between the ages of 12 and 14 years, and 81.6% had a family history of premenstrual symptoms. Nearly half of them reported academic performance impairments because of premenstrual symptoms. About 35.1% indicated that they experienced moderate-to-severe PMS, with 9.5% of the sample reporting symptoms that were consistent with PMDD (Table 1).

**Table 1:** Sample characteristics (N =  516).

Variables	Categories	Total (%)
**Age**	**Mean ± SD** (21.71 ± 10.85)	
**Age (years)**	≤21	250 (48.4)
	22 - 24	241 (46.7)
	≥25	25 (4.8)
**Religion**	Muslim	431 (83.5)
	Hindu	85 (16.5)
**Marital status**	Single	468 (90.7)
	Married	48 (9.3)
**Age at menarche (years)**	12 - 14	364 (70.5)
	≥15	41 (7.9)
	Don’t remember	24 (4.7)
	9 -11	87 (16.9)
**Family history of premenstrual symptoms**	Yes	95 (18.4)
	No	421 (81.6)
**Academic performance impairment due to premenstrual symptoms**	Yes	248 (48.1)
	No	268 (51.9)
**Chronic disease**	Yes	64 (12.4)
	No	452 (87.6)
**Physical exercise**	Yes	161 (31.2)
	No	355 (68.8)
**PMDD-PMS status**	Moderate/severe PMS	181 (35.1)
	PMDD	49 (9.5)
	No symptoms	286 (55.4)
**Depression**	Normal	162 (31.4)
	Mild	78 (15.1)
	Moderate	113 (21.9)
	Severe	95 (18.4)
	Extremely severe	68 (13.2)
**Anxiety**	Normal	176 (34.1)
	Mild	44 (8.5)
	Moderate	150 (29.1)
	Severe	55 (10.7)
	Extremely severe	91 (17.6)
**Stress**	Normal	262 (50.8)
	Mild	70 (13.6)
	Moderate	83 (16.1)
	Severe	75 (14.5)
	Extremely severe	26(5.0)

### Prevalence of suicidal ideation and suicide attempts

**Fig 1:**
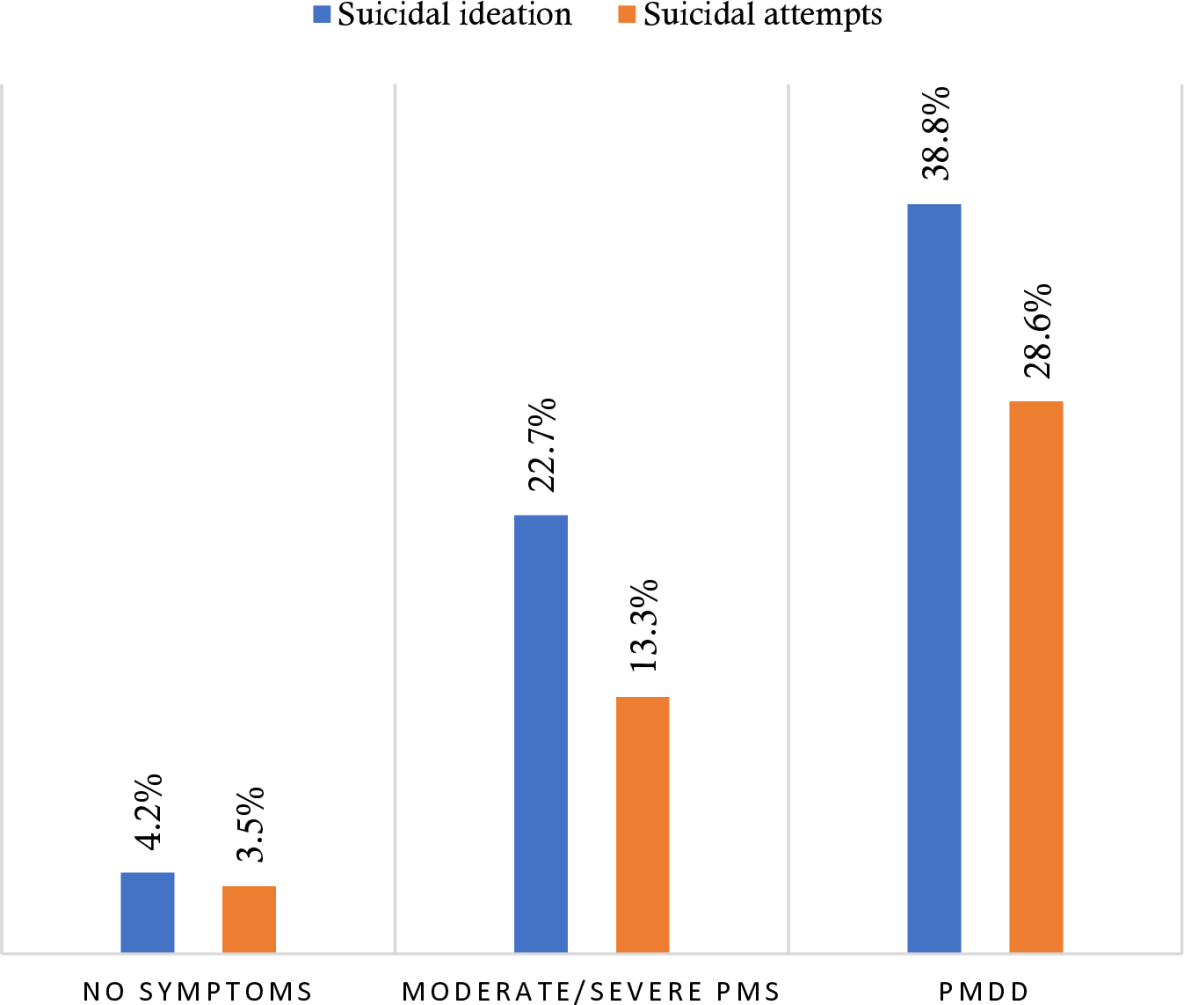
Prevalence of suicidal ideation and suicidal attempts according to premenstrual dysphoric disorder status.

In the present study, the prevalence of suicidal ideation was 13.9% (72), whereas that of suicide attempts was 9.3% (Table 2). The results indicated that among students, the prevalence of suicidal ideation was 4.2% (12) for those with no symptoms, 22.7% (41) for those with moderate/severe PMS, and 38.8% (19) for those with PMDD. Similarly, the prevalence of suicide attempts was 3.5% (10) in those with no symptoms, 13.3% (24) in those with moderate/severe PMS, and 28.6% (14) in those with PMDD (Fig 1).

**Table 2:** Distribution of sample characteristics with Past-year suicidal ideation and attempts (N =  516).

Variables	Categories	Suicidal ideation				Suicidal attempts			
		No (%)	Yes (%)	χ^2^	*p*-value	No (%)	Yes (%)	χ^2^	*p*-value
**Age**	≤21	217 (86.8)	33 (13.2)	0.27	0.874	225 (90.0)	25 (10.0)	0.65	0.723
	22 - 24	206 (85.5)	35 (14.5)			221 (91.7)	20 (8.3)		
	≥25	21 (84.0)	4 (16.0)			22 (88.0)	3 (12.0)		
**Religion**	Muslim	371 (86.1)	60 (13.9)	0.01	0.962	392 (91.0)	39 (9.0)	0.20	0.655
	Hindu	73 (85.9)	12 (14.1)			76 (89.4)	9 (10.6)		
**Marital status**	Single	401 (85.7)	67 (14.3)	0.55	0.458	425 (90.8)	43 (9.2)	0.08	0.780
	Married	43 (89.6)	5 (10.4)			43 (89.6)	5 (10.4)		
**Age at menarche (years)**	12 - 14	322 (88.5)	42 (11.5)	9.54	**0.023**	337 (92.6)	27 (7.4)	6.37	0.095
	≥15	36 (87.8)	5 (12.2)			37 (90.2)	4 (9.8)		
	9 -11	66(75.9)	21 (24.1)			74 (85.1)	13 (14.9)		
	Don’t remember	20 (83.3)	4 (16.7)			20 (83.3)	4 (16.7)		
**Family history of premenstrual symptoms**	Yes	72 (75.8)	23 (24.2)	10.20	**0.001**	72 (75.8)	23 (24.2)	30.67	**<0.001**
	No	372 (88.4)	49 (11.6)			396 (94.1)	25 (5.9)		
**Academic performance impairment due to premenstrual symptoms**	Yes	197 (79.4)	51 (20.6)	17.38	**<0.001**	212 (85.5)	36 (14.5)	15.39	**<0.001**
	No	247 (92.2)	21 (7.8)			256 (95.5)	12 (4.5)		
**Chronic disease**	Yes	45 (70.3)	19 (29.7)	15.06	**<0.001**	46 (71.9)	18 (28.1)	30.68	**<0.001**
	No	399 (88.3)	53 (11.7)			422 (93.4)	30 (6.6)		
**Physical exercise**	Yes	136 (84.5)	25 (15.5)	0.48	0.487	141 (87.6)	20 (12.4)	2.70	0.100
	No	308 (86.8)	47 (13.2)			327 (92.1)	28 (7.9)		
**PMDD-PMS**	Moderate/severe PMS	140 (77.3)	41 (22.7)	59.23	**<0.001**	157 (86.7)	24 (13.3)	36.35	**<0.001**
	PMDD	30 (61.2)	19 (38.8)			35 (71.4)	14 (28.6)		
	No symptoms	274 (95.8)	12 (4.2)			276 (96.5)	10 (3.5)		
**Depression**	Normal	157 (96.9)	5 (3.1)	90.50	**<0.001**	158 (97.5)	4 (2.5)	53.01	**<0.001**
	Mild	74 (94.9)	4 (5.1)			73 (93.6)	5 (6.4)		
	Moderate	100 (88.5)	13 (11.5)			103 (91.2)	10 (8.8)		
	Severe	78 (82.1)	17 (17.9)			88 (92.6)	7 (7.4)		
	Extremely severe	35 (51.5)	33 (48.5)			46 (67.6)	22 (32.4)		
**Anxiety**	Normal	172 (97.7)	4 (2.3)	62.47	**<0.001**	174 (98.9)	2 (1.1)	51.15	**<0.001**
	Mild	41 (93.2)	3 (6.8)			39 (88.6)	5 (11.4)		
	Moderate	130 (86.7)	20 (13.3)			140 (93.3)	10 (6.7)		
	Severe	43 (78.2)	12 (21.8)			49 (89.1)	6 (10.9)		
	Extremely severe	58 (63.7)	33 (36.3)			66 (72.5)	25 (27.5)		
**Stress**	Normal	255 (97.3)	7 (2.7)	84.19	**<0.001**	255 (97.3)	7 (2.7)	34.01	**<0.001**
	Mild	61 (87.1)	9 (12.9)			62 (88.6)	8 (11.4)		
	Moderate	63 (75.9)	20 (24.1)			71 (85.5)	12 (14.5)		
	Severe	53 (70.7)	22 (29.3)			61 (81.3)	14 (18.7)		
	Extremely severe	12 (46.2)	14 (53.8)			19 (73.1)	7 (26.9)		

### Association between different explanatory variables and the occurrence of suicidal ideation and suicide attempts

Table 2 presents the results of the relationship between different factors and the prevalence of suicidal ideations. These factors encompassed early onset of menarche (specifically between 9 and 11 years of age), which showed a significant association (χ2 = 9.54, p = 0.023), as well as a history of PMS (χ2 = 10.20, p = 0.001), academic performance decline due to PMS (χ2 = 17.38, p < 0.001), presence of chronic disease (χ2 = 15.06, p < 0.001), and presence of PMDD (χ2 = 59.23, p < 0.001). The study also revealed a strong link between suicidal ideation and self-reported high levels of depression (χ2 = 90.50, p < 0.001), anxiety (χ2 = 62.47, p < 0.001), and stress (χ2 = 84.19, p < 0.001) among the participants. Moreover, the findings indicated a significant correlation between suicide attempts and PMS (χ2 = 30.67, p < 0.001), academic performance decline due to PMS (χ2 = 15.39, p < 0.001), chronic diseases (χ2 = 30.68, p < 0.001), and PMDD (χ2 = 36.35, p < 0.001) in the student population. Lastly, there was a noteworthy association between suicide attempts and the presence of depression (χ2 = 53.01, p < 0.001), anxiety (χ2 = 51.15, p < 0.001), and stress (χ2 = 34.01, p < 0.001), according to the study’s results.

### Predictors of suicidal ideation and suicide attempts

The odds ratios for the explanatory variables in the adjusted model, presented in Table 3, provide insights into predicting the probability of suicidal ideation. Our findings indicate a significant link between experiencing moderate to severe PMS and PMDD and an increased risk of suicidal ideation among students. Compared to students without any symptoms, those with moderate-to-severe PMS had a higher likelihood of suicidal ideation (AOR =  4.73, 95% CI =  2.08–10.73), as did those with PMDD (AOR =  5.42, 95% CI =  2.02–14.53). On the other hand, students who did not have depression, anxiety, or stress exhibited a lower likelihood of having suicidal ideation (AOR =  0.17, 95% CI =  0.04–0.64), (AOR =  0.24, 95% CI =  0.06–0.89), and (AOR =  0.17, 95% CI =  0.04–0.69), respectively. Our study findings also revealed that individuals who experienced menarche between the ages of 12–14 years had a lower likelihood of suicidal ideation than those who experienced menarche at an earlier age (AOR =  0.29, 95% CI =  0.12–0.67). Additionally, a familial background of PMS was significantly associated with an increased susceptibility to suicidal ideation among student populations (AOR =  2.52, 95% CI =  1.18–5.38). Engaging in physical exercise was found to reduce the likelihood of experiencing suicidal ideation among students compared with those who did not exercise (AOR =  2.06, 95% CI =  1.02–4.16).

**Table 3:** Logistic regression analysis of the variables with Past-year suicidal ideation.

Variables	Categories	Suicidal ideation				
		Unadjusted model		Adjusted model (Nagelkerke’s R^2^ = 0.460)		VIF
		*p*-value	COR 95% CI [LL-UL]	*p*-value	AOR 95% CI [LL-UL]	
**PMDD-PMS**	Moderate/severe PMS	**<0.001**	6.69 [3.41 - 13.13]	**<0.001**	4.73 [2.08 - 10.73]	1.235
	PMDD	**<0.001**	14.46 [6.40 - 32.67]	**0.001**	5.42 [2.02 - 14.52]	
	No symptoms	Ref.		Ref.		
**Depression**	Normal	**<0.001**	0.03 [0.01 - 0.09]	**0.009**	0.17 [0.04 - 0.64]	1.898
	Mild	**<0.001**	0.06 [0.02 - 0.17]	**0.002**	0.09 [0.02 - 0.41]	
	Moderate	**<0.001**	0.14 [0.07 - 0.29]	**0.005**	0.24 [0.09 - 0.65]	
	Severe	**<0.001**	0.23 [0.11 - 0.47]	**0.002**	0.23 [0.09 - 0.58]	
	Extremely severe	Ref.		Ref.		
**Anxiety**	Normal	**<0.001**	0.04 [0.01 - 0.12]	**0.034**	0.24 [0.06 - 0.89]	1.704
	Mild	**0.001**	0.13 [0.04 - 0.45]	0.320	0.48 [0.11 - 2.03]	
	Moderate	**<0.001**	0.27 [0.14 - 0.51]	0.206	0.58 [0.24 - 1.35]	
	Severe	0.070	0.49 [0.23 - 1.06]	0.333	0.61 [0.23 - 1.66]	
	Extremely severe	Ref.		Ref.		
**Stress**	Normal	**<0.001**	0.02 [0.01 - 0.07]	**0.013**	0.17 [0.04 - 0.69]	1.981
	Mild	**<0.001**	0.13 [0.04 - 0.36]	0.468	0.60 [0.16 - 2.35]	
	Moderate	**0.006**	0.27 [0.11 - 0.68]	0.509	0.68 [0.21 - 2.16]	
	Severe	**0.027**	0.36 [0.14 - 0.89]	0.499	0.68 [0.22 - 2.11]	
	Extremely severe	Ref.		Ref.		
**Age**	<21	0.696	0.80 [0.26 - 2.47]	0.186	0.37 [0.08 - 1.62]	1.133
	22 - 24	0.843	0.89 [0.29 - 2.75]	0.368	0.51 [0.12 - 2.19]	
	≥25	Ref.		Ref.		
**Religion**	Muslim	0.962	0.98 [0.50 - 1.92]	0.244	0.61 [0.26 - 1.41]	1.030
	Hindu	Ref.		Ref.		
**Marital status**	Single	0.460	1.44 [0.55 - 3.76]	0.075	3.01 [0.90 - 10.08]	1.105
	Married	Ref.		Ref.		
**Age at menarche (years)**	12 - 14	**0.003**	0.41 [0.23 - 0.74]	**0.004**	0.29 [0.12 - 0.67]	1.028
	≥15	0.124	0.44 [0.15 - 1.26]	0.057	0.27 [0.07 - 1.04]	
	Don’t remember	0.441	0.63 [0.19 - 2.05]	0.159	0.30 [0.05 - 1.61]	
	9 -11	Ref.		Ref.		
**Family history of premenstrual symptoms**	Yes	**0.002**	2.43 [1.39 - 4.23]	**0.017**	2.52 [1.18 - 5.38]	1.124
	No	Ref.		Ref.		
**Academic performance impairment due to premenstrual symptoms**	Yes	**<0.001**	3.04 [1.77 - 5.23]	0.630	0.83 [0.40 - 1.75]	1.291
	No	Ref.		Ref.		
**Chronic disease**	Yes	**<0.001**	3.18 [1.73 - 5.84]	0.248	1.65 [0.71 - 3.83]	1.053
	No	Ref.		Ref.		
**Physical exercise**	Yes	0.487	1.20 [0.71 - 2.04]	**0.043**	2.06 [1.02 - 4.16]	1.043
	No	Ref.		Ref.		

*AOR =  Adjusted odds ratio, COR =  Crude odds ratio, LL =  Lower limit, UL =  Upper limit, VIF =  Variation Inflation Factor*

The findings presented in Table 4 demonstrate a strong link between the presence of moderate to severe PMS and PMDD symptoms among students and an increased likelihood of attempting suicide. Students with moderate to severe PMS had higher odds of suicide attempts (AOR =  3.77, CI =  1.36–10.50), as did those with PMDD (AOR =  4.07, 95% CI =  1.22–13.56), than students without any symptoms. Conversely, the absence of depression and anxiety decreased the probability of contemplating suicide attempts (AOR =  0.16, 95% CI =  0.03–0.84) and (AOR =  0.09, 95% CI =  0.02–0.59), respectively. Our analysis also revealed that students who experienced menarche between the ages of 12–14 had a significantly reduced likelihood of engaging in suicide attempts (AOR =  0.33, 95% CI =  0.12–0.91) compared to those who experienced menarche between the ages of 9–11. Furthermore, our study found a significant association between familial predisposition to PMS among student populations and an increased likelihood of attempting suicide (AOR =  7.39, 95% CI =  2.99–18.25). Additionally, patients with chronic diseases had significantly higher odds of suicide attempts than those without chronic diseases (AOR =  3.10, 95% CI =  1.20–7.99, p =  0.019). Finally, engaging in physical exercise was associated with increased odds of suicide attempts (AOR =  3.83, 95% CI =  1.59–9.26, p =  0.003).

**Table 4:** Logistic regression analysis of the variables with Past-year suicidal attempts.

Variables	Categories	Suicidal attempts				
		Unadjusted model			Adjusted model (Nagelkerke’s R^2^ = 0.470)	VIF
		*p*-value	COR 95% CI [LL-UL]	*p*-value	AOR 95% CI [LL-UL]	
**PMDD-PMS**	Moderate/severe PMS	**<0.001**	4.22 [1.97 - 9.05]	**0.011**	3.77 [1.36 - 10.50]	1.235
	PMDD	**<0.001**	11.04 [4.56 - 26.73]	**0.023**	4.07 [1.22 - 13.56]	
	No symptoms	Ref.		Ref.		
**Depression**	Normal	**<0.001**	0.05 [0.02 - 0.16]	**0.031**	0.16 [0.03 - 0.84]	1.898
	Mild	**<0.001**	0.14 [0.05 - 0.40]	0.056	0.22 [0.05 - 1.04]	
	Moderate	**<0.001**	0.20 [0.09 - 0.46]	**0.028**	0.25 [0.07 - 0.86]	
	Severe	**<0.001**	0.17 [0.07 - 0.42]	**0.001**	0.09 [0.02 - 0.36]	
	Extremely severe	Ref.		Ref.		
**Anxiety**	Normal	**<0.001**	0.03 [0.01 - 0.13]	**0.012**	0.09 [0.02 - 0.59]	1.704
	Mild	**0.041**	0.34 [0.12 - 0.96]	0.728	1.27 [0.33 - 4.89]	
	Moderate	**<0.001**	0.19 [0.09 - 0.42]	**0.005**	0.20 [0.06 - 0.61]	
	Severe	**0.022**	0.32 [0.12 - 0.85]	0.345	0.55 [0.16 - 1.91]	
	Extremely severe	Ref.		Ref.		
**Stress**	Normal	**<0.001**	0.07 [0.02 - 0.23]	0.827	0.82 [0.15 - 4.65]	1.981
	Mild	0.071	0.35 [0.11 - 1.09]	0.198	3.06 [0.56 - 16.79]	
	Moderate	0.150	0.46 [0.16 - 1.33]	0.986	0.99 [0.22 - 4.39]	
	Severe	0.374	0.62 [0.22 - 1.77]	0.608	1.45 [0.35 - 6.04]	
	Extremely severe	Ref.		Ref.		
**Age**	<21	0.753	0.81 [0.23 - 2.92]	0.673	1.50 [0.23 - 9.79]	1.133
	22 - 24	0.533	0.66 [0.18 - 2.41]	0.974	1.03 [0.16 - 6.68]	
	≥25	Ref.		Ref.		
**Religion**	Islam	0.656	0.84 [0.39 - 1.81]	0.081	0.38 [0.13 - 1.13]	1.030
	Hindu	Ref.		Ref.		
**Marital status**	Single	0.780	0.87 [0.33 - 2.31]	0.888	0.91 [0.23 - 3.59]	1.105
	Married	Ref.		Ref.		
**Age at menarche (years)**	12 - 14	**0.030**	0.46 [0.22 - 0.93]	**0.032**	0.33 [0.12 - 0.91]	1.028
	≥15	0.423	0.62 [0.19 - 2.02]	0.372	0.49 [0.10 - 2.36]	
	Don’t remember	0.836	1.14 [0.33 - 3.87]	0.599	0.61 [0.10 - 3.79]	
	9 -11	Ref.		Ref.		
**Family history of premenstrual symptoms**	Yes	**<0.001**	5.06 [2.72 - 9.40]	**<0.001**	7.39 [2.99 - 18.25]	1.124
	No	Ref.		Ref.		
**Academic performance impairment due to premenstrual symptoms**	Yes	**<0.001**	3.62 [1.84 - 7.14]	0.568	1.32 [0.51 - 3.44]	1.291
	No	Ref.		Ref.		
**Chronic disease**	Yes	**<0.001**	5.50 [2.85 - 10.64]	**0.019**	3.10 [1.20 - 7.99]	1.053
	No	Ref.		Ref.		
**Physical exercise**	Yes	0.103	1.66 [0.90 - 3.04]	**0.003**	3.83 [1.59 - 9.26]	1.043
	No	Ref.		Ref.		

*AOR =  Adjusted odds ratio, COR =  Crude odds ratio, LL =  Lower limit, UL =  Upper limit, VIF =  Variation Inflation Factor*

### Interaction effects of depression, anxiety, and stress on the relationships between PMS/PMDD, suicidal ideation, and suicide attempts

**Fig 2:**
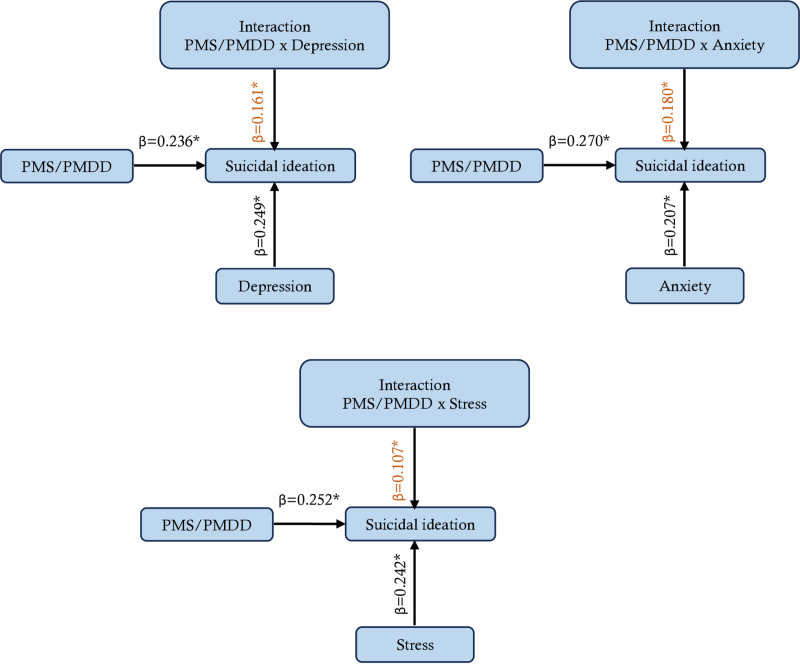
Interaction effects of PMS/PMDD with depression, anxiety, and stress on suicidal ideation.

The results presented in Fig 2 demonstrate a significant relationship between suicidal ideation and PMS/PMDD (β =  0.236, 0.270, and 0.252; p <  0.05). Suicidal ideation was predicted by depression (β =  0.249, p <  0.05), anxiety (β =  0.207, p <  0.05), and stress (β =  0.242, p <  0.05). Significant interaction effects were observed between depression (β =  0.161, p <  0.05), anxiety (β =  0.180, p <  0.05), tension (β =  0.107, p <  0.05) and PMS/PMDD.

**Fig 3:**
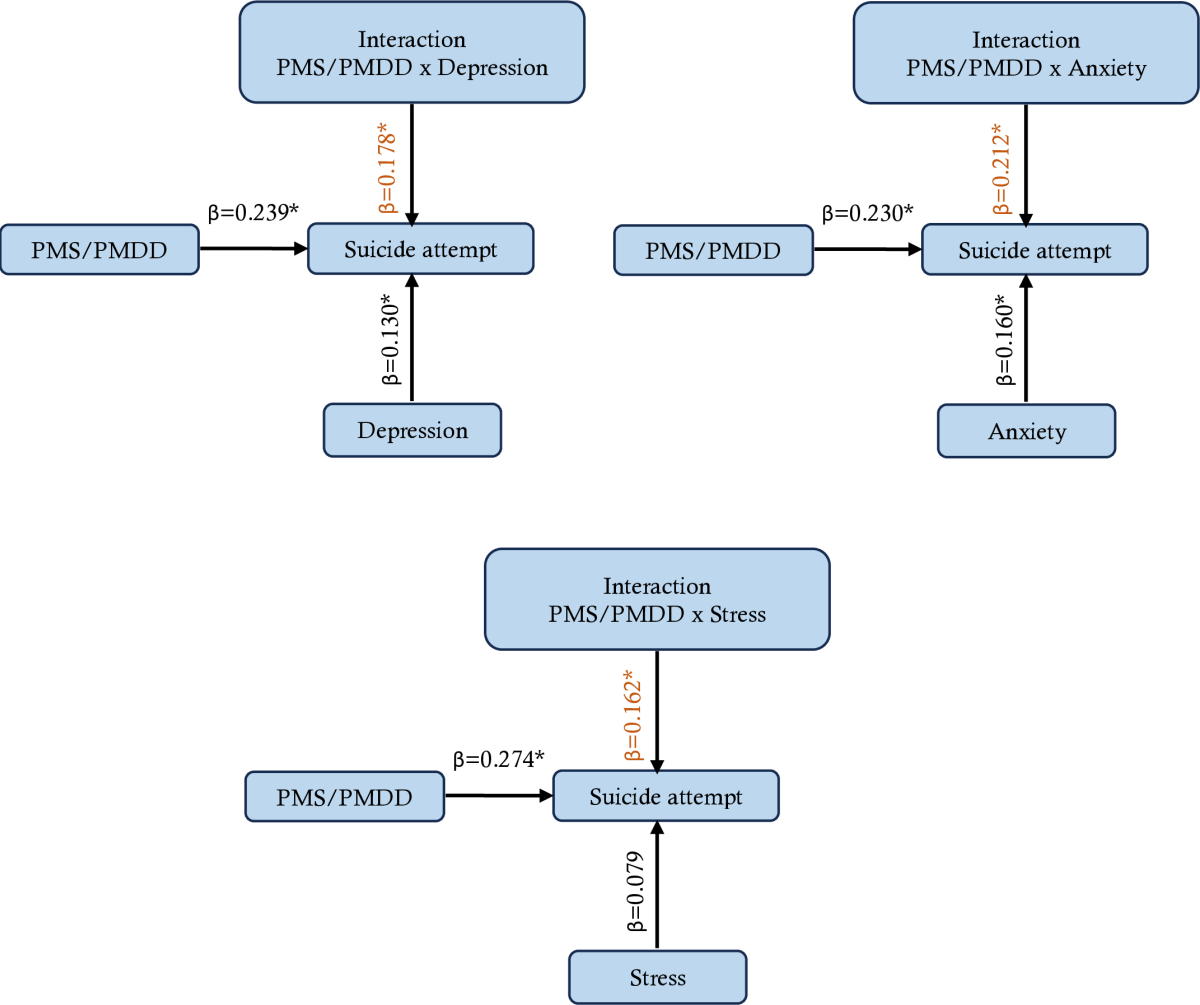
Interaction effects of PMS/PMDD with depression, anxiety, and stress on suicide attempt.

Additionally, the findings showed a strong correlation between suicide attempts and PMS/PMDD (β =  0.239, 0.230, and 0.274, respectively; p <  0.05) (Fig 3). Stress did not exhibit a significant direct influence (β =  0.079, p >  0.05), depression (β =  0.130, p <  0.05) and anxiety (β =  0.160, p <  0.05) independently predicted suicide attempts. PMS/PMDD was found to have significant interaction effects with stress (β =  0.162, p <  0.05), anxiety (β =  0.212, p <  0.05), and depression (β =  0.178, p <  0.05).

## Discussion

The objective of this study was to examine the possible association between the presence of PMDD and suicidal behaviors, specifically suicidal thoughts and suicide attempts, among a group of students from Bangladesh. To our knowledge, this is the first known examination of this association in this population. The present investigation yielded findings indicating that the occurrence of past-year suicidal ideation among students who exhibited no symptoms, moderate/severe PMS, and PMDD was 12 (4.2%), 41 (22.7%), and 19 (38.8%), respectively. The prevalence of past-year suicide attempts was 10 (3.5%), 24 (13.3%), and 14 (28.6%), respectively. The findings of this study are of particular significance as they indicate a positive association between the severity of PMS and PMDD and the likelihood of engaging in suicidal behavior. This relationship follows a dose-response pattern, whereby higher levels of PMS and PMDD are linked to an elevated risk of suicidal tendencies.

Similar to the present study, a secondary data analysis was conducted on a cohort of 3,965 females from the United States [34]. Their findings revealed that among women who reported the absence of symptoms, moderate/severe PMS, and PMDD, the rates of suicidal thoughts were 13.3%, 22.0%, and 37.4%, respectively [34]. However, the rates of suicide attempts among individuals without symptoms, moderate/severe PMS, and PMDD were 4.9%, 7.4%, and 16.2%, respectively, which were lower than the rates observed in the current study [34]. In another study involving adolescents from the United States and Nigeria, notable levels of suicidal ideation were observed among those who reported the absence of symptoms, moderate/severe PMS, and PMDD, with prevalence rates of 10.9%, 24.4%, and 18.2%, respectively [35]. Additionally, Hong et al. (2012) found that individuals with a history of PMDD exhibited significantly higher rates of suicidal ideation (45.8% vs. 17.3%), suicide plans (16.9% vs. 4.2%), and suicide attempts (13.6% vs. 3.9%) than those without PMDD [36]. The observed disparity could potentially be ascribed to multiple variables, such as variances within the sampled demographics, sociocultural differentiation, and the implementation of diverse assessment instruments to measure the dependent variables.

Six studies were conducted to explore the potential correlation between PMDD and a history of suicide attempts, as well as the occurrence of current suicide attempts [34,35,37–40]. Consistent with the findings of our investigation, the majority of the studies (five out of six) demonstrated a noteworthy association between PMDD and instances of suicide attempts. However, one study observed a significant association solely in the unadjusted analysis, but upon accounting for potential confounding factors such as age and psychiatric disorders, the association lost its statistical significance [35]. Furthermore, it is worth noting that four separate studies have examined the correlation between PMDD and the presence of suicidal ideation [34,35,38,41]. In line with the current study, it is interesting that three out of four studies demonstrated an association between PMDD and suicidal ideation [34,35,41]. Moreover, eight studies were conducted to evaluate the correlation between PMS and a history of suicide attempts or ongoing suicide attempts [34,37,39,42–46]. Among the studies examined, it is notable that three of them have identified an association between PMS and suicide attempts, which aligns with the findings of our study [42,44,46]. Conversely, five studies reported no significant relationship between these variables [34,37,39,43,45]. Five studies were conducted to evaluate the potential correlation between PMS and the occurrence of suicidal ideation [34,37,39,43,45]. Consistent with our findings, all five studies have identified a statistically significant correlation between PMS and the occurrence of suicidal ideation. Despite variations in some methodologies, our findings regarding the link between PMDD and suicidality were consistent with previous research. However, the current body of evidence is constrained by its reliance on cross-sectional or retrospective case-control study designs, which warrants further study to establish a clear causal relationship between PMDD and suicidal behavior.

The existing body of research has not examined the specific mechanisms that connect PMDD with suicidality. Evidence suggests that serotonin dysfunction is a critical factor in both PMDD and suicidal behavior. Research indicates that individuals with PMDD often exhibit impairments in serotonergic function, which is also observed in individuals with suicidal tendencies [27,47]. Specifically, low serotonin levels have been correlated with increased suicidal behavior, highlighting the neurotransmitter’s role in mood regulation and impulsivity [27,47]. This connection is further supported by findings that women with severe PMDD experience heightened emotional dysregulation, which may exacerbate their vulnerability to suicidal behaviors [27,47].

Moreover, fluctuations in estrogen and progesterone levels during the menstrual cycle have a major impact on mood and behavior, especially in women who are prone to PMS and PMDD. These hormonal changes are linked to the hypothalamic-pituitary-adrenal (HPA) axis, which regulates cortisol levels and the body’s stress response [48]. The HPA axis is an important component of the body’s stress response, and disruption is frequently observed in people with mood disorders. In fact, research has shown that women with PMDD may have altered HPA axis activity, which results in aberrant cortisol responses to stress [49]. Elevated cortisol levels, which are frequently associated with chronic stress, can contribute to mood dysregulation and increase the risk of suicidal behavior [50]. Therefore, the interaction between serotonin deficiencies, hormonal changes, and HPA axis dysregulation emphasizes the complex neurobiological pathways underlying PMDD and its association with suicidality.

In addition, our findings showed that PMS/PMDD, anxiety, depression, and stress had significant interaction effects. These results imply that the degree of concurrent anxiety, depression, and stress symptoms affects the association between PMS/PMDD and suicidal thoughts rather than just being additive. For example, a systematic review by Prasad et al., women with PMDD are more likely to have suicidal thoughts, especially if they also have mental comorbidities [27]. Findings by Gao et al. support this, showing that women with PMDD are at a heightened risk of suicidality, especially if they also have bipolar or anxiety disorders [51]. Interestingly, although stress did not significantly affect suicide attempts directly, our results suggest that stress strongly interacts with anxiety, depression, and PMS/PMDD to predict suicide attempts. This implies that the association between PMS/PMDD and suicidal actions may be moderated by stress, an aspect that warrants further investigation. This argument is supported by the literature, which shows that stress can exacerbate the emotional dysregulation linked to PMDD, raising the risk of suicidal ideation and attempts [52]. These results of interaction effects suggest that women with PMS/PMDD are more likely to experience suicidality as their anxiety, depression, and stress symptoms worsen, underscoring the importance of thorough mental health evaluation in this population.

The results of our study indicate a notable correlation between early onset of menstruation and the occurrence of suicidal behavior, even after accounting for various confounding factors, such as depression, anxiety, and stress. These findings suggest that the onset of menarche may trigger the release of gonadal hormones, which could potentially impact the development of the brain, thereby increasing susceptibility to psychopathology [53] The findings indicate a potential association between factors, such as increased biological susceptibility to suicidal behavior resulting from early exposure to sex hormones. Females who undergo early menarche exhibit a swift transformation in their physical appearance as a means of preparing for reproductive behavior. As a result of these alterations, individuals may experience heightened levels of stress to modify their way of life, coupled with increased levels of apprehension and shame compared to their counterparts [54]. Females who experience early onset of menarche may exhibit physical maturation but may not necessarily exhibit concurrent emotional maturation. As a result, they may be susceptible to adverse outcomes such as depression [55]. Individuals may employ risk-taking cognitive strategies when confronted with stressful life events. This may be attributed to less developed cognitive maturation in comparison to physical maturation, which could hinder their capacity to adjust to changes in various domains relative to their peers. Additionally, their self-regulatory ability may be diminished [54]. The potential correlation between premature physical development and delayed emotional/cognitive development could be linked to suicidal behavior among females who experienced early onset of menstruation.

The present study has several notable strengths that warrant attention. The current study employed the PSST, a thoroughly validated and all-encompassing tool, to assess the extent of PMS severity. This study represents a novel investigation of the potential association between PMDD status and various suicidal behaviors among a sample of university students in Bangladesh. Previous research on this subject has focused on analyzing clinical samples or samples that are relatively homogeneous. In our study, we implemented appropriate measures to account for demographic and psychiatric factors that may be associated with suicidal behavior. This was done to accurately determine the impact of the PMDD status on such behaviors. Previous research has failed to consider a diverse array of potential confounding or mediating factors.

### Implications of our findings

The findings emphasize the urgent need for integrated mental and menstrual health therapies for female university students, particularly those with PMDD or severe PMS, who are considerably more likely to report suicide conduct. Expanding campus mental health programs to address mild to moderate depression, anxiety, and stress may help reduce suicidal thoughts and prevent it from progressing to severe psychological distress. The vulnerability of early-maturing students was addressed, emphasizing the importance of early adolescent mental health support services. The association between a family history of PMS and suicidal ideation suggests a genetic or learned behavioral component, which justifies family-inclusive mental health education campaigns. Although not significant, the data indicated that academic impairment caused by PMS/PMDD and chronic conditions may not directly predict suicidal ideation, stressing the importance of comprehensive student support services. Physical exercise is a protective factor, highlighting the importance of regular physical activity as part of female students’ mental health measures. These findings support comprehensive campus health initiatives that include menstrual health education, mental health services, and lifestyle interventions to lower the risk of suicide and promote student well-being. The presence of PMS/PMDD alongside depression, anxiety, and stress increases the risk of suicide, emphasizing the need for university health centers to implement focused mental health programs that treat the combined impact of PMS/PMDD and associated psychological illnesses.

### Limitations

The present study exhibits several constraints, indicating potential directions for future investigation. It is important to note that because of the retrospective nature of the data collection and the lack of clinician interviews, precise diagnoses may not have been attainable. Additionally, the study lacked a mechanism for ascertaining the presence of a premenstrual exacerbation, which could potentially amplify an underlying psychiatric or medical condition. This study did not distinguish between women who reported no PMS symptoms and those who reported mild PMS symptoms. It should be noted that the present study was cross-sectional; therefore, only the factors associated with the outcome were identified. As such, it was not possible to establish a causal relationship between the variables under investigation. Additionally, the use of convenience sampling may result in selection bias, reducing the external validity and generalizability of the results to a broader population. Future studies using probability sampling methods may produce more representative results and improve the robustness of their findings. The study’s findings should be interpreted considering the potential social desirability bias introduced by face-to-face interviews (for questions like suicidal ideation and suicide attempts) despite mitigation efforts. It is widely acknowledged in the literature that the manifestations of PMS and PMDD tend to arise during the luteal phase. The use of a cross-sectional design for the PSST in this investigation may have resulted in the confounding of PMS subgroups owing to the presence of participants who experienced comparable discomfort in other phases. The DASS-21 scale, which is valid for assessing depression, anxiety, and stress, is a limitation of this study. It lacks the depth necessary for clinical diagnosis, which could result in the overlooked severity of mental health conditions. Moreover, the investigation did not evaluate the potential interactions between PMS/PMDD and suicidal ideation, which could be influenced by other psychiatric comorbidities, including personality disorders, PTSD, or bipolar disorder. The internal validity of the findings may be compromised by these factors, as they were not considered during the analysis. Future research should consider the utilization of more comprehensive diagnostic instruments and the inclusion of a broader spectrum of psychiatric conditions. The study did not address menstrual cycle abnormalities and medication use, including hormonal treatments such as oral contraceptives, which may affect the interpretation of their influence on mental health outcomes.

## Conclusions

In summary, a substantial percentage of women who manifested PMDD, as evaluated using a validated assessment tool, exhibited a significant prevalence of past-year suicidal ideation and suicide attempts. In accordance with the outcomes of a recent systematic review, it is advisable to acknowledge women diagnosed with PMDD as a demographic with an elevated susceptibility to suicidal tendencies. The findings of this study suggest that healthcare practitioners tasked with the provision of care for individuals diagnosed with PMDD should undertake a comprehensive assessment of their patients to identify potential signs of suicidal behavior. Furthermore, healthcare professionals must consider the implementation of therapeutic interventions for PMS in females who manifest indications of suicidal ideation, plans, or attempts. By directing attention towards a sex-specific risk factors, healthcare practitioners possess the potential to address a noteworthy public health concern that affects women within the reproductive age group, particularly those encountering PMS or PMDD, as well as individuals who have undergone suicide attempts but survived. Routine mental health examinations should be integrated into normal healthcare services to detect women with PMS or PMDD as well as co-existing depression, anxiety, and stress. These comprehensive strategies can considerably enhance the overall well-being and quality of life of women with PMS/PMDD and related mental health issues.

## Supporting information

S1 Dataset Dataset for the present study. (XLSX)
